# Hemosiderotic dermatofibroma mimicking melanoma in a 12‐year‐old boy: a case report

**DOI:** 10.1002/ccr3.1508

**Published:** 2018-04-06

**Authors:** Emine Müge Acar, Murat Tad, Asuman Kilitci, Funda Kemeriz

**Affiliations:** ^1^ Department of Dermatology Kırşehir Ahi Evran University Training and Research Hospital Kırşehir 40200 Turkey; ^2^ Department of Pathology Kırşehir Ahi Evran University Training and Research Hospital Kırşehir 40200 Turkey; ^3^ Department of Dermatology Aksaray University Training and Research Hospital Aksaray 68200 Turkey

**Keywords:** Child, dermoscopy, hemosiderotic dermatofibroma, melanoma

## Abstract

We report a case of hemosiderotic dermatofibroma presenting as a brown‐black‐colored nodule with peripheral extensions, which mimics melanoma. Histopathology showed completely benign features with no atypia or mitosis. Nodular extensions of childhood dermatofibromas may be related to the growth of the child not necessarily pointing to a malignant process.

## Introduction

Dermatofibroma (DF) is a common benign fibrohistiocytic tumor of the skin and has a slight female preponderance 1. DF is characterized by solitary or multiple, firm, red‐brown‐colored dermal nodules with a predilection for the lower extremities 2, 3. DF most commonly affects young adults and can also be seen in pediatric age group 1, 3, 4. The diagnosis of the classical type of dermatofibroma is predominantly based on the clinical and dermoscopic features, but atypical variants can pose a diagnostic challenge 1, 5, 6. Hemosiderotic DF is an atypical and rare variant of DF that can be clinically and dermoscopically indistinguishable from melanoma 3. Here, we report a case of hemosiderotic DF, which clinically mimics melanoma, on the right knee of a 12‐year‐old boy.

## Case Report

**Figure 1:**
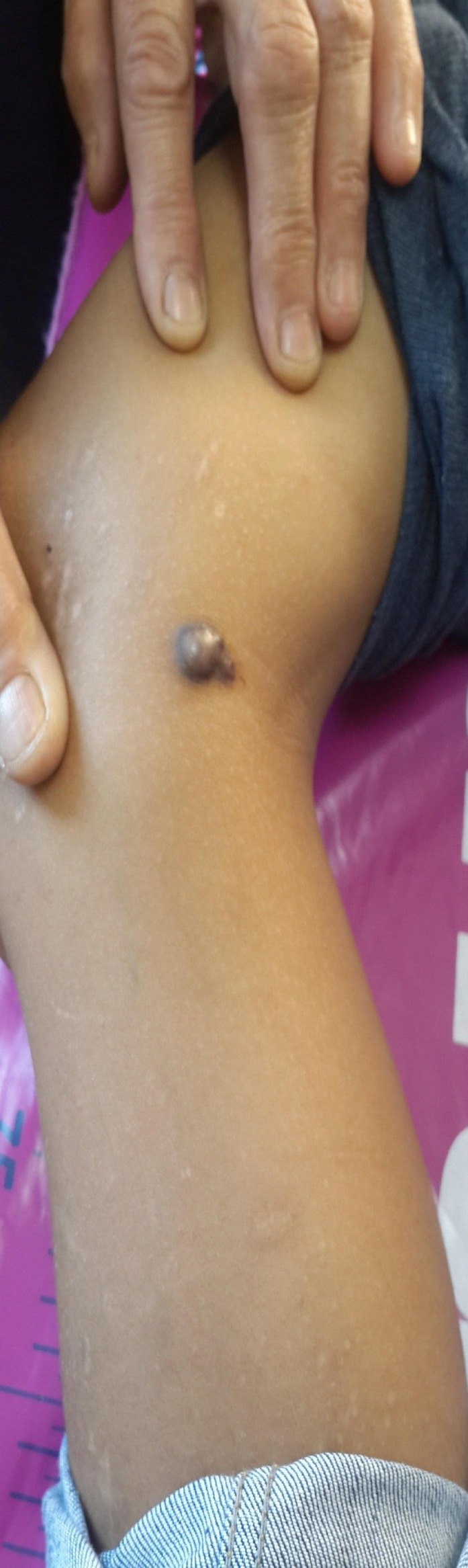
Dark brown‐black nodule (1 × 1 cm) on the medial aspect of the leg.

**Figure 2:**
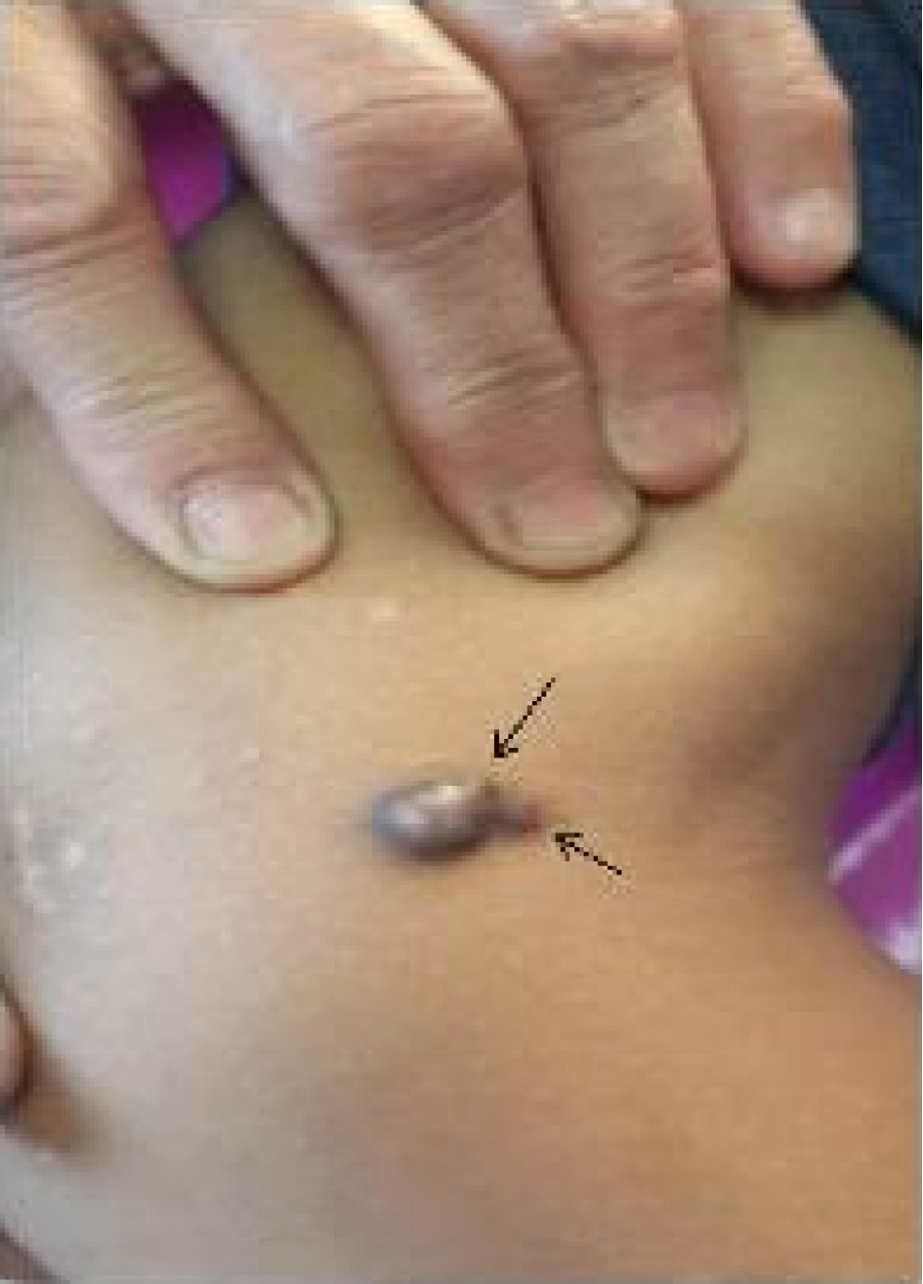
Nodular extensions toward popliteal fossa on close image (black arrows).

A 12‐year‐old boy presented to our clinic with a firm, nodular mass on the medial aspect of the right knee with a history of about 1 year. The lesion was not pruritic or painful, but mild tenderness was present. The patient stated that the size of the nodule had gradually increased over the last 3 months. Physical examination revealed a dark brown‐black nodule, measuring about 1 × 1 cm, with two small nodular extensions from the periphery (Figs 1 and 2). There was neither ulceration nor crusting, and the dimple sign was negative. The patient gave no history of skin disease or other systemic disorders.

**Figure 3:**
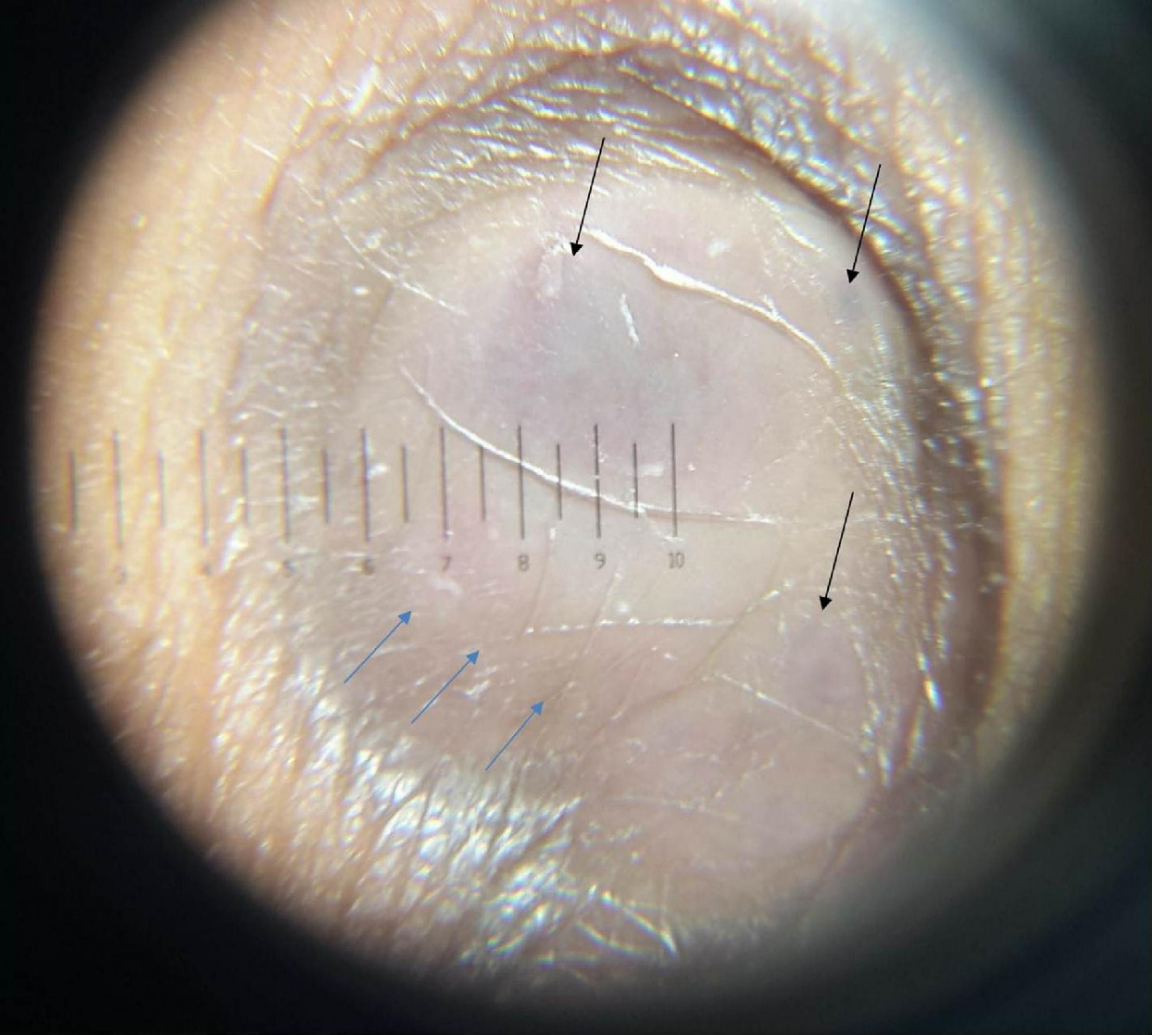
Dermoscopic image with blue‐red pigmentation areas on a white structureless area (black arrows) and pinkish hue at the periphery (blue arrow) surrounded by brown pigmentation.

**Figure 4:**
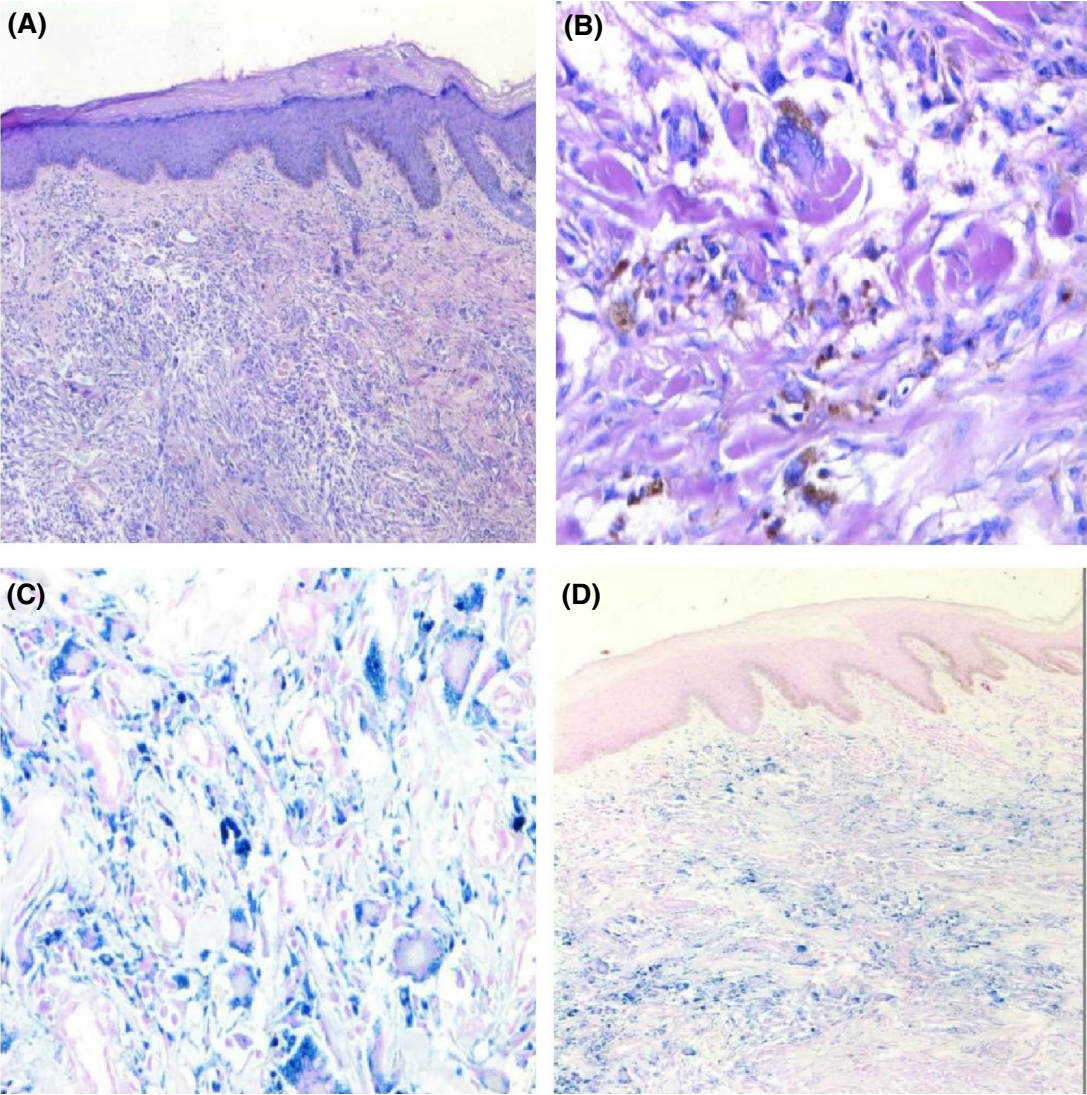
Histopathological examination (A, B): Interlacing fascicles of spindle cells in dermis with a storiform pattern, intracellular and extracellular hemosiderin deposits (H&E,X50; H&E,X100); (C, D): Positive histochemical staining of Prussian blue, showing hemosiderin deposits, in most of the spindle‐shaped cells (X100;X50).

Dermoscopy revealed focally localized blue‐red pigmentation areas on a white structureless background and pinkish red area at the periphery surrounded by brown pigmentation (Fig. 3). The lesion was excised with 5‐mm clear margins with preliminary diagnosis of nodular melanoma. On histopathological examination, a benign tumor composed of interlacing fascicles of spindle cells in the dermis with a storiform pattern and containing hemosiderin deposits was seen (Fig. 4A and B). Intracellular and extracellular hemosiderin deposits were demonstrated with the Prussian blue stain (Fig. 4C and D). Atypia and mitosis were not present. Factor XIIIa (FXIIIa) staining was positive, and CD34, S‐100, smooth muscle actin (SMA), and cytokeratin (AEI/AEI3) stains were negative. The diagnosis of hemosiderotic DF was established with these histopathological findings. There was no recurrence during 1 year of follow‐ up.

## Discussion

Dermatofibroma is a benign dermal fibrohistiocytic tumor with a slight female predominance 1. It is generally seen in young adults and is less often reported in children 3. Most DFs present in the lower extremities as slowly growing solitary nodules. In our patient, the lesion was localized on the leg which has a predilection for dermatofibroma.

The common variant of DF is characterized by a well‐circumscribed, red‐brown nodule typically located on the legs 2, 3. In our patient, the lesion presented as a round, dark‐brown, black‐colored nodule. The nodule showed two nodular extensions to the periphery, developing from the lesion toward the popliteal fossa. This was a unique feature as dermatofibromas with peripheral extensions and has not been previously reported to our knowledge. Sehgal et al. 7 reported a giant DF with satellite lesions around the dermatofibroma plaque. However, in our patient, these small nodules grew directly from the DF and gave an impression of an actively growing lesion, as seen in cutaneous malignancies and mainly nodular melanoma. Rapid enlargement in the last 3 months and the dark brown‐black color of the lesion also strongly supported this provisional diagnosis. As the lesion was found to be consistent with dermatofibroma, the peripheral extension was considered to be a feature pointing to the lesion's evolution in this case.

DFs can display a variety of dermoscopic patterns. A peripheral delicate pigment network and central white scar‐like patch is the typical and most common dermoscopic feature of DF 8. Zaballos et al. 9 reported that most hemosiderotic and aneurysmal DFs showed multicomponent pattern including a central bluish or reddish homogenous areas and peripheral delicate network with vascular structures of variable degrees. Kilinc et al. 2 reported focal blue‐gray pigmentation and a peripheral pinkish hue in two hemosiderotic DFs. We similarly observed, focal blue‐red areas on a white structureless area and a focal pinkish red hue at the periphery, with blue pigmented areas most likely representing the proliferation of hemosiderin‐containing macrophages in our patient.

Hemosiderotic DF is considered as a precursor stage of aneurysmal DF by some authors 6. Aneurysmal DF can present with a blue‐brown nodule that can rapidly grow due to extensive hemorrhage and therefore pose a diagnostic challenge with melanocytic and vascular lesions. In the histopathological examination of our patient, Prussian blue demonstrated intracellular and extracellular hemosiderin deposition. Atypia, mitosis, or necrosis were not present. HMB 45 and Melan A, which are markers for melanoma, were negative. CD34 was positive; other markers such as S‐100, SMA, cytokeratin AEI/AEI3, and FXIIIa stains were negative and were used to exclude other mesenchymal neoplasms.

Dermatofibroma is generally known as a benign mesenchymal neoplasm of the skin, but high cellularity, increased atypia, and proliferative activity can be seen histopathologically especially in the cellular, aneurysmal, and atypical variants 1. These variants may have an aggressive growth pattern and are more likely to metastasize. In a study by Mentzel et al. 10 comprising seven cases of DF with recurrences and metastasis, chromosomal aberrations were detected in five patients by array comparative genomic hybridization (CGH). CGH may be a helpful technique in the recognition of DFs with malignant potential. Histopathological features such as high proliferative activity and increased atypia were not detected in our patient, and we therefore did not perform CGH or further examination despite the peripheral spreading of the nodule leading to a suspicion of a malignant lesion clinically. Simple excision is generally curative for DFs, and the local recurrence rate is <2% 1. The lesion in our patient was excised with 5‐mm margins, and no recurrence was seen after 1 year of follow‐up.

Hemosiderotic DF, which is a rare variant of dermatofibroma, has never been reported in children until now as far as we are aware. Our case was also interesting as the lesion clinically gave the impression of a rapidly growing melanoma with its dark brown‐black color and peripheral spreading, but no features pointing to a malignancy were detected on histopathological examination, thus forming a clinicopathological discordance. The clinical appearance was attributed to the DF lesion's evolution with the growth of the patient. It must be kept in mind that DFs can enlarge with peripheric nodular extensions in childhood, not necessarily pointing to a malignant process. Our case also reveals that hemosiderotic DFs must be considered in the differential diagnosis of pigmented nodular lesions in children.

## Conflict of Interest

The authors declare that there is no conflict of interest regarding the publication of this article.

## Authorship

EMA: involved in the acquisition of clinical and histopathological data, design and draft of the manuscript, and revision and approval of the final manuscript; MT and AK: interpreted the histopathological data, designed and drafted the manuscript, and approved the final manuscript; FK: designed and drafted the manuscript, and approved the final manuscript.
